# Falls, fracture and frailty risk in multiple sclerosis: a Mendelian Randomization study to identify shared genetics

**DOI:** 10.1007/s00774-024-01504-8

**Published:** 2024-05-27

**Authors:** Sohyun Jeong, Ming-Ju Tsai, Changbing Shen, Yi-Hsiang Hsu

**Affiliations:** 1 Hinda and Arthur Marcus Institution for Aging Research, Hebrew SeniorLife, Boston, MA 02131 USA; 2 Department of Medicine, Beth Israel Deaconess Medical Center and Harvard Medical School, Boston, MA 02215 USA; 3 Department of Dermatology, Peking University Shenzhen Hospital, Shenzhen, 518036 Guangdong China; 4 Shenzhen Key Laboratory for Translational Medicine of Dermatology, Shenzhen Peking University - The Hong Kong University of Science and Technology Medical Center, Shenzhen, 518036 Guangdong China; 5 Broad Institute of MIT and Harvard, Cambridge, MA USA

**Keywords:** Multiple sclerosis, Fracture, Frailty, Falls, Mendelian randomization

## Abstract

**Introduction:**

Patients with multiple sclerosis (MS) commonly present musculoskeletal disorders characterized by lower bone mineral density (BMD) and muscle weakness. However, the underlying etiology remains unclear. Our objective is to identify shared pleiotropic genetic effects and estimate the causal relationship between MS and musculoskeletal disorders.

**Materials and Methods:**

We conducted linkage disequilibrium score regression (LDSR), colocalization, and Mendelian randomization (MR) analyses using summary statistics from recent large-scale genome-wide association studies (GWAS), encompassing MS, falls, fractures, and frailty. Additional MR analyses explored the causal relationship with musculoskeletal risk factors, such as BMD, lean mass, grip strength, and vitamin D.

**Results:**

We observed a moderate genetic correlation between MS and falls (RG = 0.10, P-value = 0.01) but not between MS with fracture or frailty in the LDSR analyses. MR revealed MS had no causal association with fracture and frailty but a moderate association with falls (OR: 1.004, FDR q-value = 0.018). We further performed colocalization analyses using nine SNPs that exhibited significant associations with both MS and falls in MR. Two SNPs (rs7731626 on ANKRD55 and rs701006 on OS9 gene) showed higher posterior probability of colocalization (PP.H4 = 0.927), suggesting potential pleiotropic effects between MS and falls. The nine genes are associated with central nervous system development and inflammation signaling pathways.

**Conclusion:**

We found potential pleiotropic genetic effects between MS and falls. However, our analysis did not reveal a causal relationship between MS and increased risks of falls, fractures, or frailty. This suggests that the musculoskeletal disorders frequently reported in MS patients in clinical studies are more likely attributed to secondary factors associated with disease progression and treatment, rather than being directly caused by MS itself.

**Supplementary Information:**

The online version contains supplementary material available at 10.1007/s00774-024-01504-8.

## Introduction

Multiple sclerosis (MS) is a demyelinating inflammatory autoimmune disorder characterized by continuous and diffuse changes in the white and grey matter, and damage to axons [1]. Despite recent advancements in disease-modifying therapies, patients with MS still exhibit an increased risk of musculoskeletal disorders including incidents of accidents and falls [2], fractures [3, 4], and frailty [5] when compared to unaffected controls.

Falls represent a debilitating consequence of MS, with as many as 56% of MS patients experiencing falls in any 3-month period [2]. A meta-analysis encompassing nine cohort studies suggests 1.58 times increased risk of fractures among patients with MS in comparison to their non-affected counterparts [6]. Frailty is an indicator of poor prognosis in patients suffering from systemic sclerosis [5] and a significantly higher percentage of MS patients display frailty in comparison to the control group, with percentages of 28% and 8%, respectively [5].

Multiple risk factors for developing musculoskeletal disorders in patients with MS have been proposed. These include systemic bone loss by chronic inflammatory status [7], muscle weakness due to immobility [8], ataxia from disruption of neuronal impulses [9], and bone and muscle loss due to MS treatment such as corticosteroid use [10]. In addition to these secondary factors, intrinsic elements of MS etiology may also be associated with increased musculoskeletal disorders. For instance, even early-stage comparatively young MS patients with minimal or no physical disability also displayed low bone mineral density (BMD) [11].

However, most of the findings were typical observational studies not eliminating potential biases from undefined residual confounding and reverse causation. Consequently, gaining a deeper understanding of the impact of genetically unmodifiable predisposing factors will provide valuable insights into the care of patients with MS.

To address this, we implemented LD score regression, colocalization and multiple Mendelian Randomization (MR) approaches to estimate the genetic pleiotropic effect and direct MS effects to 3 musculoskeletal outcomes (fracture, falls, and frailty) adjusting for the confounding effects from well-established bone and muscle disorder-related predisposing factors such as bone mineral density (BMD), lean body mass (whole body fat-free mass, appendicular lean mass), grip strengths and vitamin D levels.

We performed the following analyses: (1) Linkage Disequilibrium Score Regression (LDSR) to assess genetic heritability and genetic pleiotropy (correlations) between MS and 3 musculoskeletal traits (falls, fracture, and frailty) and predisposing factors; (2) Univariable and multivariable MR to assess the causal effect of MS on falls, fracture, and frailty, and heel estimated BMD (eBMD), whole body fat-free mass, appendicular lean mass, grip strengths and vitamin D levels; (3) Bidirectional MR to assess the directionality among fracture, falls, frailty; (4) Colocalization analysis to delineate shared genetic variants.

## Materials and Methods

### Linkage Disequilibrium Score Regression (LDSR)

LDSR can estimate cross-trait genetic correlations using GWAS summary statistics data [12]. We conducted LDSR analyses with the LD Hub interface [13] and the LDSC package (https://github.com/bulik/ldsc). We restricted our LDSR analyses to well-imputed SNPs (imputation quality score > 0.8) and SNPs with Minor Allele Frequency (MAF) ≥ 5% in the study populations. In addition, because outliers can unduly influence the regression, we also excluded SNPs with extremely large effect sizes (X_1_^2^ > 80) [14]. The MS GWAS (47,429 MS cases and 68,374 non-MS healthy controls) summary statistics was obtained from the recent MS GWAS meta-analysis of the International Multiple Sclerosis Genetics Consortium (IMSGC) [15]. As for other musculoskeletal traits, we used GWAS summary statistics from the UK Biobank with the LD hub pipeline to estimate genetic heritability and assess correlations with MS.

### Mendelian Randomization (MR)

MR is a widely used method using measured variation in genes (genotypes, SNPs) of exposure to examine the causal effect of exposures on disease outcomes in observational studies [16, 17].

Most GWAS summary statistics data were downloaded from the “MRC IEU OpenGWAS database” repository (https://gwas.mrcieu.ac.uk/) or each GWAS consortia.

**Fig. 1:**
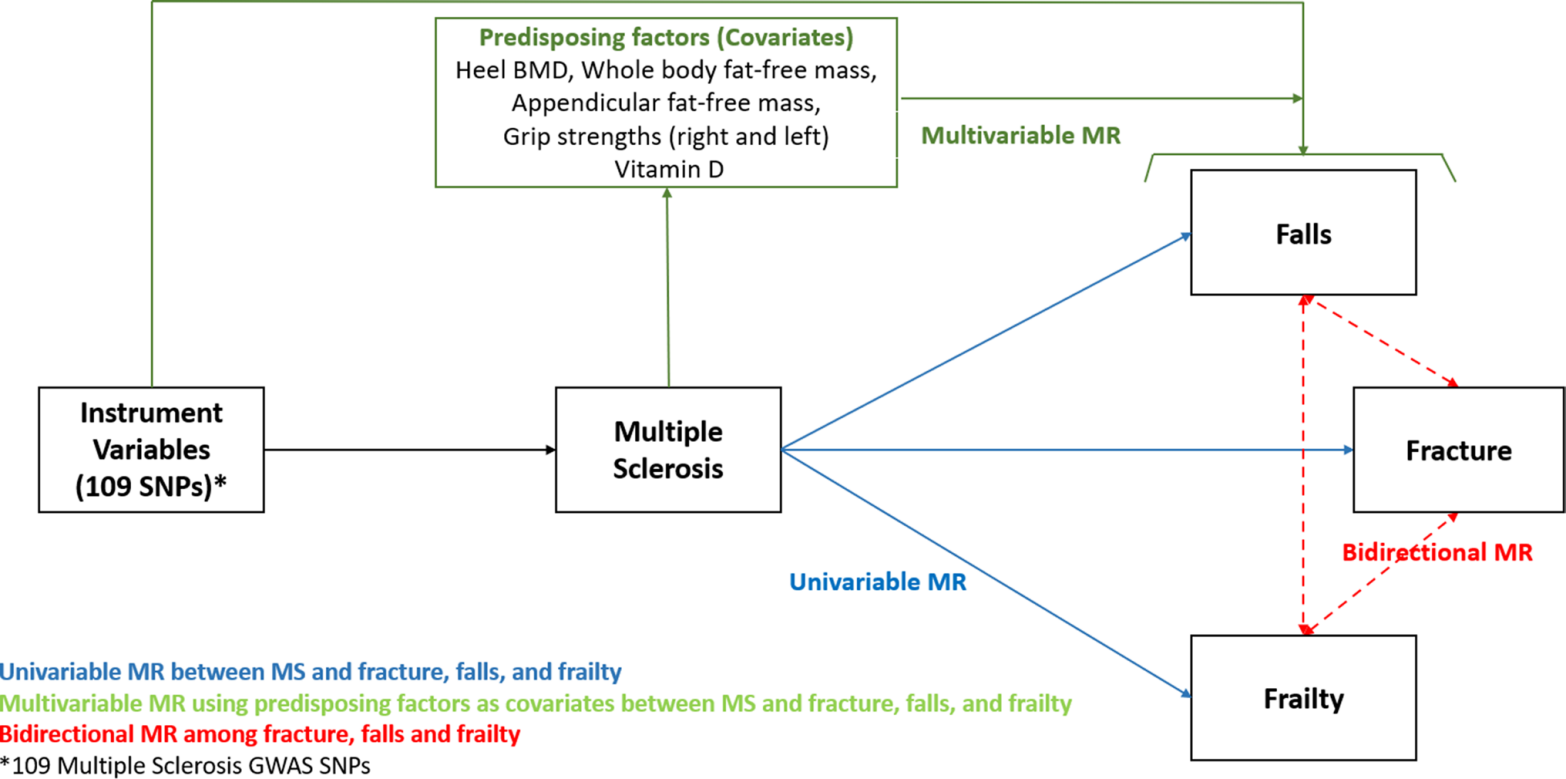
Conceptual study designs and MR analyses with univariable, multivariable, and bidirectional MR models

The information on diagnostic criteria, phenotype definition, sample size, name of consortium, and dataset IDs from the database are available in Supplementary Table 1. The overall study design for MR is displayed in Fig. 1.

#### Exposure data

Instrumental variables (IVs) for MS were obtained from the recent GWAS meta-analysis of the IMSGC [15]. The detailed analysis and results description are provided in the previous IMSGC publication [15]. The estimated MS heritability is ~ 48%. Among the genome-wide significantly associated SNPs (*p*-values ≤ 5 × 10^–8^), 200 SNPs are located in the autosomal and non-MHC genomic region. Among these 200 SNPs, we further excluded 91 SNPs in LD with the lead SNPs. A total of 109 genome-wide associated SNPs were selected as IVs for MR analysis. The Effect allele, reference allele, effect allele frequency, and effect size information on these 109 SNPs are summarized in Supplementary Table 2. The minor alleles of these 109 SNPs are all larger than 10% in study populations. The effect size (OR) ranges from 1.06 to 1.23.

#### Outcome data

Fracture and fall GWAS data used in the analysis were selected from the UK Biobank data, fractured/broken in the last 5 years (ID: ukb-b-13346, n = 44,502 cases and 415,887 controls) [18] and falls last year (ID: ukb-b-2535, n = 461,725). Frailty GWAS data were obtained from a study by Atkins et al. in 2019 (n = 175,226) [19].

#### Predisposing factors of musculoskeletal outcomes

Known bone and muscle strength-related factors were used as covariates in multivariable MR (MVMR). They are heel-estimated BMD (eBMD) [19], appendicular lean mass, whole body fat-free mass, right handgrip strength, left grip strength [20] vitamin D [21]. These covariates were considered in MVMR to control for their potential confounding effects on musculoskeletal three outcomes.

### Statistical analysis

#### Univariable MR between MS and three musculoskeletal traits as well as predisposing factors

Univariable MR was applied to assess the causal relationship between MS and 3 musculoskeletal outcomes (fracture, falls, and frailty) and 5 predisposing factors. We additionally performed MR between 5 predisposing factors and 3 musculoskeletal outcomes to examine potential underlying pathways between them. Multi-instrument MR strategy was applied. This approach involves conducting single instrumental variable MR analyses for each exposure or risk factor independently, and then the MR results from these multiple instrumental variables were combined through a fixed-effect meta-analysis. This meta-analysis allowed us to estimate the overall causal relationships between the exposures (MS and predisposing factors), and musculoskeletal outcomes.

#### Multivariable MR (MVMR) between MS and three musculoskeletal outcomes adjusting for predisposing factors

We also applied regression-based MVMR to account for many variants having pleiotropic effects that are associated with multiple predisposing factors. This method provides coefficients or effect sizes that represent the direct causal effects of multiple sclerosis (MS) on three distinct musculoskeletal outcomes while holding other predisposing factors constant. The estimation of causal effects in MVMR involves performing regression analyses of the associations between genetic variants and the outcome of interest on the associations between the same genetic variants and the selected predisposing factors. The intercept in these regression analyses is set to zero, and the weights used are the inverse variances of the associations with the outcome [22]. Predisposing factors that demonstrated significant associations in the univariable MR analysis with each musculoskeletal trait were included in the MVMR analysis.

#### Bidirectional MR among the three musculoskeletal outcomes

Bidirectional MR was performed among fracture, falls, and frailty to examine their mutual effects and directionality considering their highly correlated nature. In bidirectional MR, instruments for both exposure and outcome are used to evaluate whether the “exposure” variable causes the “outcome” or whether the “outcome” variable causes the “exposure”[23].

#### Heterogeneity analysis

We employed Cochran’s Q statistic as a diagnostic tool to assess the heterogeneity of IVs. Heterogeneity can indicate a potential violation of the necessary assumptions for MR or the presence of pleiotropic effects, where a single genetic variant influences multiple traits. Cochran’s Q statistic derived from the inverse variance weighted (IVW) estimate should follow a χ^2^ distribution with degrees of freedom equal to the number of SNPs minus 1. When there is excessive heterogeneity, it suggests that either the modeling assumptions for MR have been violated or that some of the genetic variants are not adhering to the MR assumptions [24]. This is termed ‘horizontal pleiotropy’[25]. When Q statistics showed significant heterogeneity, we applied the MR-PRESSO [26] method to reassess the MR effects after filtering out outlier SNPs that may be responsible for the observed heterogeneity. After removing these outliers, we compared the MR results obtained from the MR-PRESSO adjusted analysis with the fixed-effects MR results derived from the IVW approach.

#### Sensitivity analysis

The sensitivity analysis including IVW, weighted median, weighted mode, and MR-Egger tests was performed in univariable MR analysis. For MVMR, IVW and MR-Egger tests were evaluated. We also applied the MR-Egger regression test [27] to verify horizontal pleiotropy in each MR. Among the multiple methods, we set the results from IVW and outlier removal results from MR-PRESSO as primary findings.

All the MR analyses were conducted in R (version 4.0.0) and specialized packages, including TwoSampleMR [28], MendelianRandomization [29], MRPRESSO [30], and the MR-Base platform [31]. The causal effect size (beta or OR) with standard error and p-values were presented as appropriately. We applied a false discovery rate (FDR, *q*) to account for multiple testing corrections. The criteria used to select final instrument variables in each MR were GWAS significance: *p* < *5e*^*−8*^, LD clumping: r^2^ > 0.001 within a 10 MB window, and proxy SNPs in 1000 Genomes EUR, r^2^ = 0.8 [32].

### Colocalization

We performed colocalization analysis to identify genetic variants that were associated with both multiple sclerosis (MS) and falls as potential pleiotropic genetic effects. The R COLOC package (https://chr1swallace.github.io/coloc/index.html) was used [33]. The R COLOC package calculates approximate Bayes Factors, which help quantify the evidence for colocalized genetic variants between two traits. We focused on examining a 500 kb region around each site (250 kb on either side) [34] specifically targeting nine SNPs that displayed a significant association in the MR analysis between MS and falls. The posterior probabilities of H0 (no causal variant), H1 (causal variant for trait 1 only), H2 (causal variant for trait 2 only), H3 (two distinct causal variants), and H4 (one common causal variant) are calculated. Among their output results, PP (Post-probability > 95%) H4 was used to determine the high probability of colocalized genetic variants between 2 traits. Conversely low PP4 (< 50%) means we cannot identify which individual SNP is jointly causal with confidence [35].

## Results

### Genetic heritability and correlations by LDSR

LDSR was performed using genetic information from 1,293,150 single nucleotide polymorphisms (SNPs) in the MS GWAS dataset. After merging with the reference panel for LD, a total of 1,109,876 SNPs remained for the final analysis. LDSR demonstrated that MS exhibits a statistically significant moderate genetic correlation with falls (rG: 0.105, *P-value* = *0.010*) but not with fracture (rG: − 0.017, *P-value* = *0.711*) or frailty (rG: 0.151, *P-value* = *0.082*). Additionally, both handgrip strengths and both leg fat-free masses also showed a genetically significant correlation with MS. In contrast, the whole-body fat-free mass did not exhibit a significant genetic correlation with MS. The heritability of these traits estimated from GWAS summary statistics was low or considered not high; fracture (*h*^*2*^:0.019), falls (*h*^*2*^: 0.033), and frailty (*h*^*2*^: 0.109) (Table 1).

**Table 1:** LD score, heritability, and genetic correlation information between MS and musculoskeletal phenotypes

	LD score				Heritability	Genetic correlation**			
	Mean χ^2^	λ_GC_	Intercept (se)*	Intercept Z	h^2^ (se)	rG (se)	rG_Z	rG*_p*	Intercept (se)
Multiple Sclerosis	1.319	1.151	1.009 (0.008)	1.594	0.101 (0.008)	Ref	ref	ref-	ref
Fractured/broken bones in last 5 years	1.130	1.113	1.007 (0.008)	1.033	0.019 (0.002)	− 0.017 (0.047)	− 0.371	0.711	− 0.003 (0.005)
Falls in the last year	1.218	1.195	1.004 (0.008)	1.947	0.033 (0.002)	0.105 (0.041)	2.591	**0.010**	− 0.004 (0.005)
Frailty	1.394	1.320	1.056 (0.009)	1.219	0.109 (0.005)	0.151 (0.087)	1.741	0.082	0.001 (0.006)
Heel eBMD	2.233	1.507	1.080 (0.035)	1.823	0.297 (0.033)	− 0.003 (0.025)	− 0.132	0.895	− 0.002 (0.007)
Whole body fat-free mass	3.030	2.161	1.114 (0.029)	6.643	0.291 (0.012)	0.035 (0.025)	1.395	0.163	− 0.001 (0.008)
Hand grip strength (R)	1.744	1.553	1.058 (0.013)	5.749	0.104 (0.004)	− 0.066 (0.027)	− 2.454	**0.014**	0.002 (0.006)
Hand grip strength (L)	1.742	1.558	1.055 (0.012)	5.751	0.104 (0.004)	− 0.067 (0.028)	− 2.427	**0.015**	0.002 (0.006)
Leg fat-free mass (R)	2.846	2.096	1.099 (0.026)	6.157	0.266 (0.011)	0.060 (0.025)	2.359	**0.018**	− 0.004 (0.008)
Leg fat-free mass (L)	2.849	2.108	1.092 (0.025)	5.880	0.268 (0.011)	0.062 (0.025)	2.435	**0.015**	− 0.005 (0.008)
Arm fat-free mass (R)	2.563	2.038	1.091 (0.026)	5.610	0.267 (0.011)	0.019 (0.025)	0.766	0.444	0.003 (0.007)
Arm fat-free mass (L)	2.564	2.035	1.097 (0.025)	5.619	0.267 (0.011)	0.030 (0.025)	1.191	0.234	0.002 (0.007)

$\lambda$_GC_, genomic inflation factor; h^2^,heritability: rG, genetic correlation between two traits; rG_Z, Z score of the genetic correlation; rG_p, p-value of the genetic correlation

^*^Single-trait LD Score regression intercept for each trait

^**^ Genetic correlation between Multiple sclerosis and each of the musculoskeletal traits

Significant results are highlighted in bold

### Mendelian Randomization

#### Univariable MR

**Fig. 2:**
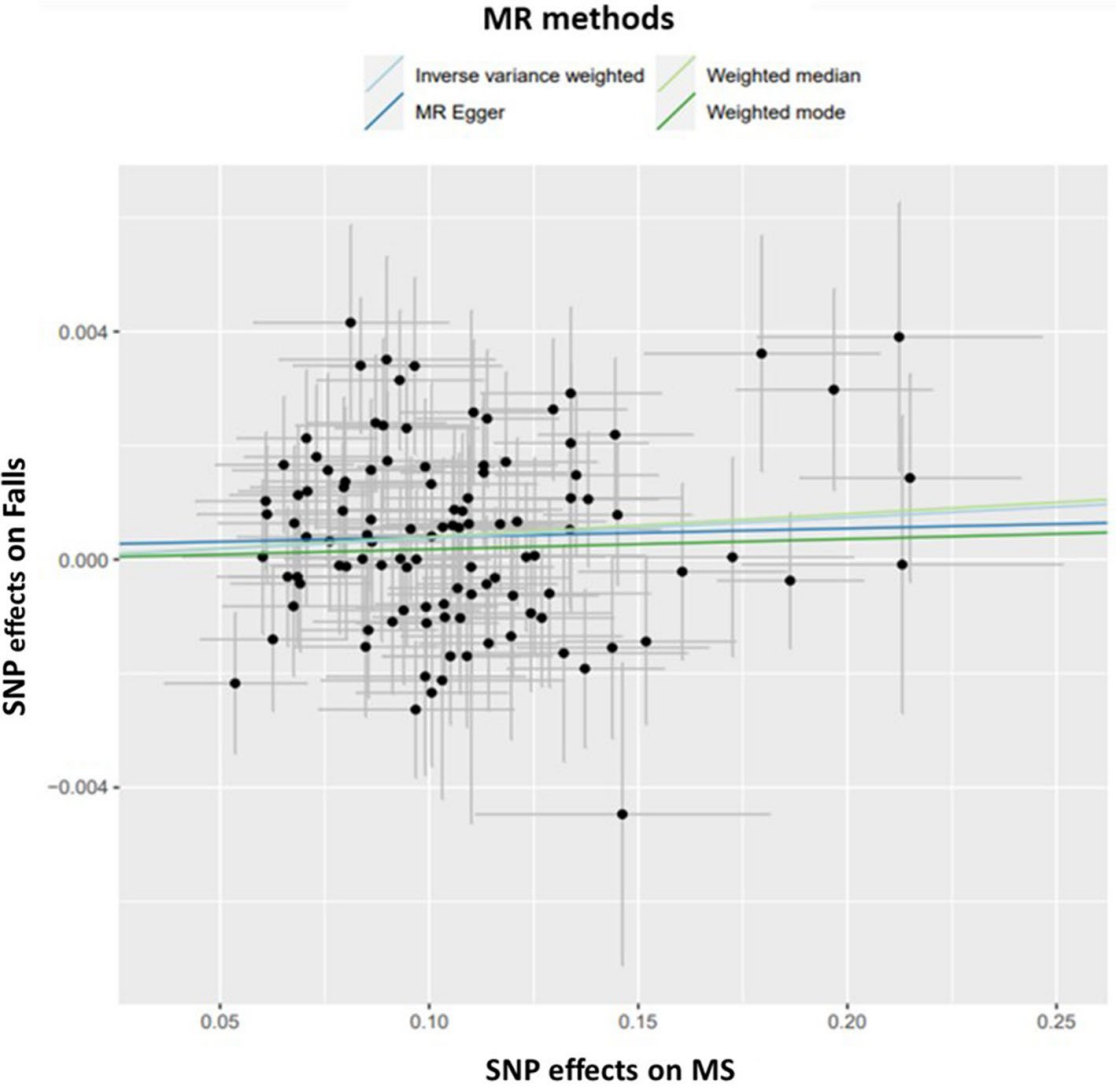
Scatter plot of univariable Mendelian Randomization effects between MS and falls

We didn’t find a significant causal relation between MS and fracture (OR: 1.002, 95% CI: 1.000–1.003, *FDR q-value* = *0.056*); as well as no significant causal relation between MS and frailty (*β*: 0.009, 95% CI: 0.001–0.017, *FDR q-value* = *0.147)* as shown in Table 2. After excluding outlier SNPs by MR-PRESSO and applying multiple testing corrections, we found a significant causal relation between MS and falls (OR: 1.004, 95% CI: 1.001–1.006, FDR *q-value* = *0.018*). However, the causal relation between MS and falls has a moderate effect size (OR: 1.004), suggesting the causal relation is just a statistical significance due to large sample size in the MR analyses and may not be clinically meaningful. In addition to IVW and MR-PRESSO two MR methods, we performed additional MR analyses based on MR-egger, Weighted median and Weighted mode, three additional MR analyses. None of these additional MR analyses showed statistical significance (*P*-value > 0.05 on Supplementary Table 3). To evaluate the potential pleiotropic effect that may violate MR test assumption, a horizontal pleiotropy test was done by the MR Egger regression with the intercept test. We didn’t find any pleiotropy effect since the directionality p-value under the horizontal pleiotropy test are all > 0.05 (Supplementary Table 3). A scatter plot of univariable MR with multiple methods is presented in Fig. 2, suggesting a weak and positive causal relation between MS and falls. A funnel plot visualizing the MR results between MS and falls for each SNP was generated (Supplementary Fig. 1). No sign of genetic pleiotropy in IVW or MR-Egger methods was observed by funnel plot.

**Table 2:** Univariable MR effects between exposure (MS) and outcomes (musculoskeletal outcomes and predisposing factors)

Exposure	Outcomes	MR effects between MS and outcomes							
		MR method	#SNP	Beta	SE	P-value	Q-value	OR	95% CI
Multiple Sclerosis	Musculoskeletal traits								
	Fracture	IVW	109	0.002	0.001	0.001		1.002	1.000–1.004
		MR-PRESSO*		0.002	0.001	0.007	0.056	1.002	1.001–1.003
	Fall	IVW	109	0.004	0.001	0.005		1.004	1.002–1.006
		MR-PRESSO		0.004	0.001	0.002	**0.018**	1.004	1.001–1.006
	Frailty (continuous)	IVW	109	0.012	0.004	0.007		-	0.004–0.020
		MR-PRESSO		0.009	0.004	0.021	0.147	-	0.001–0.017
	Musculoskeletal predisposing factors								
	Heel eBMD	IVW	109	− 0.002	0.005	0.722	0.722	–	–
	Whole body fat-free mass	IVW	109	0.004	0.004	0.258	0.722	–	–
	Appendicular fat-free mass	IVW	109	0.006	0.007	0.402	0.722	–	–
	Grip strength (right)	IVW	109	− 0.004	0.003	0.183	0.722	–	-
	Grip strength (left)	IVW	109	− 0.002	0.003	0.389	0.722	–	–
	Vitamin D	IVW	105	− 0.003	0.003	0.427	0.722	–	–

IVW, inverse variance weighted; MR-PRESSO, Mendelian Randomization Pleiotropy RESidual Sum and Outlier

eBMD, estimated Bone mineral density

No horizontal pleiotropy was found in all analysis tested by Egger regression intercept test

The Heterogeneity test (Q test) was significant in all analysis except MS to fracture MR analysis

To overcome the heterogeneity of SNPs effects, MR-PRESSO outlier tests were applied, and outlier corrected results were presented along with results from IVW method

Q-value; FDR by Benjamini–Hochberg method

^*^No outlier detected

Significant results are made in bold type (Q-value)

To further identify individual SNPs that contribute to MR analysis between MS and Falls, we performed MR analysis per SNP. Among 109 SNPs, 9 SNPs were found to be significantly associated between MS and falls (MR analysis *p*-values < 0.05 on supplementary Table 4). These 9 SNPs are rs9878602, rs7731626, rs9808753, rs10191360, rs11852059, rs1026916, rs701006, rs140522 and rs3737798 (Supplementary Table 4). Except for SNP rs10191360, all the other 8 SNPs showed positive causal relations between MS and Falls. Except for SNP rs9808753 (missense variant on the *IFNGR2* gene), all the remaining 8 SNPs are non-coding variants and located in or near *FOXP1*, *ANKRD55*, *GNNG2*, *STAT3*, *OS9*, *ODF3B* and *VANGL2  * genes. These genes are involved in several pathophysiological pathways relevant to arthritis, infection, bone cancers, rhabdomyosarcoma, migraines, and neural tube defects (Supplementary Table 5).

#### Multivariable MR

In the MVMR analysis, adjustment of each predisposing factor observed by previous studies (that were identified as risk factors of musculoskeletal diseases) did not modify the overall MR results between MS and falls (*P*-values < 0.05 on Table 3). As for causal relation between MS and fractures, comparing to the unadjusted Model (univariate MR), similar results were found from MVMR analyses adjusting for each predisposing factor (Supplementary Table 6). The same MVMR analysis was also performed between MS and frailty. Compared to the unadjusted Model (univariate MR), similar results were found from MVMR analyses adjusting for each predisposing factor (Supplementary Table 7). The MVMR results suggested there are no clinical meaningful causal associations between MS and fractures as well as between MS and frailty.

**Table 3:** Multivariable MR between MS and falls adjusting for predisposing factors of musculoskeletal disorders

	MR effects between MS and falls				
	Beta	SE	P-Value	OR	95% CI
Unadjusted model	0.004	0.001	0.005	1.004	1.002–1.006
Adjusted by frailty	0.002	0.001	0.058	1.002	1.000–1.004
Adjusted by appendicular fat free mass	0.004	0.001	**0.004**	1.004	1.002–1.006
Adjusted by whole body fat free mass	0.004	0.001	**0.004**	1.004	1.002–1.006
Adjusted by heel eBMD	0.004	0.001	**0.004**	1.004	1.002–1.006
Adjusted by right grip strength	0.003	0.001	**0.011**	1.003	1.000–1.005
Adjusted by left grip strength	0.003	0.001	**0.008**	1.003	1.000–1.005
Adjusted by all except frailty	0.003	0.001	**0.022**	1.003	1.000–1.005
Adjusted by all	0.002	0.001	0.184	1.002	1.000–1.004

eBMD, estimated Bone mineral density

Significant results are made in bold

#### Bidirectional MR among the 3 musculoskeletal outcomes

To account for the directional effects among the 3 musculoskeletal outcomes of fracture, fall, and frailty, we conducted bidirectional MR. Fall causally increased fracture risk (OR: 1.215, 95% CI: 1.098–1.345, *P-value* = *1.63E−04*) and frailty risk (OR: 4.870, 95% CI: 2.412–9.832, *P-value* = *1.01E−05*). Our results are consistent with previous findings that falls causally associated with an increased risk of fractures and frailty.

### Colocalization analysis

In colocalization analysis using those nine significantly associated SNPs between MS and falls, two of them, rs7731626 and rs140522 presented over 92% colocalization rate (SNP probability of colocalization, single SNP PP.H4 = 0.927, supplementary Table 8).

## Discussion

In this study, we identified genetic correlations with potentially shared pleiotropic effects between MS and falls proven by LDSR, colocalization, and MR. However, contrary to epidemiological observation of an increased risk of musculoskeletal traits (such as fractures and frailty) in MS patients, we did not find a meaningful causal relationship between MS and fractures as well as between MS and frailty via MR analyses. Specifically, none of the well-known muscle and bone-related predisposing factors (BMD, whole-body and appendicular fat-free masses, both grip strengths, and vitamin D) were causally affected by MS. In bidirectional MR among the three musculoskeletal outcomes (fracture, fall, and frailty), we found that falls causally increased the risk of both fracture and frailty. This observation is consistent with previous reports from many epidemiological studies; suggesting our MR analyses did have adequate statistical power and validated instrumental variables to pick up causal relation signals with the GWAS summary statistics we used. We further explored SNPs that are significantly associated with both MS and falls in a MR analysis. We identified 9 potential pleiotropic SNPs. These 9 SNPs are located in/near genes that are involved in pathophysiological pathways relevant to arthritis, infection, bone cancer, rhabdomyosarcoma, migraine, neural and tube defects. Two SNP rs7731626 and rs140522, located in/near *ANKRD5* and *OS9* genes are also confirmed by the by the colocalization analyses between MS GWAS and Falls GWAS. These two genes are associated with arthritis and bone cancer, suggesting the involvement in inflammation and autoimmunity in bone cells.

Most of the previous observational studies predominantly reported high fracture risk in MS in comparison to age-matched controls. For example, Bazelier et al. 2011 [36] reported threefold-increased hip fracture risk in patients with MS during 5 years of follow-up. In addition, the falls history was also higher in MS patients, suggesting increased fractures observed in MS patients in Bazelier et al.’s study might be due to an increased risk of falls. Falls are common in patients with MS. A large international data set demonstrated 56% MS patients fall at least once within 3 months period with 37% categorized as frequent fallers [2]. Notably, MS patients fall more frequently, experience more injurious falls, and are more likely to attribute their falls to tripping and distraction [37]. These epidemiological observations support our MR findings that MS causally increased the risk of falls.

Based on our genetic correlation, MR and colocalization analyses, it appears that genetic factors may causally increase risks for both MS and falls. However, it is important to note that the effects of these genetic factors on fall risk are relatively minimal; therefore, other non-genetic factors, such as medication effects may need to be taken into consideration. Medication use is a well-established risk factor for falls, either directly (affecting balance, attention, or muscle tone) or indirectly (as proxies of underlying conditions influencing the risk of falling; joint pain, arthrosis, and cardiovascular diseases). In particular, psychotropic drugs (antidepressants, anxiolytics, and anti-epileptics) use [38, 39] are strongly associated with falls in MS patients. A recent fall GWAS study examined the genetic correlation of falls with medication use and found medications such as opioids, anti-inflammatory and anti-rheumatic drugs and drugs for peptic ulcer and gastro-esophageal reflux disease were in positive genetic correlations with falls (40). Medication use associated with MS treatments and complications may be one of the strong risk factors for falls in MS patients.

Given the highly increased availability of public GWAS data, we could utilize LDSR, a multifaceted MR approach, and colocalization analysis together and could elucidate the causal relations and identify shared genetic pleiotropic effects to draw new perspectives on musculoskeletal traits in MS patients. However, it should be caution about the reliability and limitation of MR analyses due to the low heritability of many complex traits, including some of the musculoskeletal traits. Nevertheless, we are able to observe statistical significance causal relation between MS and falls and identify several possible shared pleiotropic genetic variants that genetically predisposed arthritis and bone cancers that might have a role in fall risk in MS patients.

In conclusion, our study does not provide evidence to support the notion that multiple sclerosis (MS) causally increases the risks of fractures and frailty. The musculoskeletal disorders frequently observed in clinical studies among MS patients may be more likely attributable to secondary factors associated with MS disease progression and its treatment. We identified genetic association between MS and falls with shared pleiotropic genetic factors.

### Supplementary Information

Below is the link to the electronic supplementary material.

Supplementary file1 (DOCX 109 KB)
