# Supplementary material for: Falls, fracture and frailty risk in multiple sclerosis: a Mendelian Randomization study to identify shared genetics
Source: J Bone Miner Metab. 2024 May 27;42(3):335–43. doi: 10.1007/s00774-024-01504-8 (PMC11147890; doi:10.1007/s00774-024-01504-8)
Supplement: Supplementary file 1 — Supplementary file1 (DOCX 109 KB) [file 774_2024_1504_MOESM1_ESM.docx]

**Supplementary Table 1. GWAS summary statistics used in MR analysis**

| **Traits** | **Sample size** | **Diagnostic criteria and phenotype definition** | **Consortium** | **First author** | **Dataset ID*** |
| --- | --- | --- | --- | --- | --- |
| Multiple sclerosis (exposure) | 47,429 cases/  68,374 controls | ICD-10 code: G35, ICD-9 code: 340 | IMSGC | Patsopoulos NA et al, 2019 | ieu-b-18 |
| **Musculoskeletal outcomes** | | | | | |
| Fractured/broken bones in last 5 years (outcome) | 44,502 cases/  415,887 controls | Answered "Yes" to UK Biobank Assessment Centre Environment (ACE) touch screen survey question "Have you fractured/broken any bones in the last 5 years?" | MRC-IEU | NA | ukb-b-13346 |
| Falls last year | 116,102 cases/  474,071 controls | Answered "Yes" to UK Biobank Assessment Centre Environment (ACE) touchscreen question "In the last year have you had any falls?" | MRC-IEU | NA | ukb-b-2535 |
| Frailty | 175,226 | Frailty index (FI) continuous values: 49 or 44 self‐reported items on symptoms, disabilities and diagnosed diseases | NA | Atkins et al, 2019 | NA |
| **Predisposing factors** | | | | | |
| Heel BMD (eBMD) | 142,487 | Bone Mineral Density (BMD) measured by quantitative ultrasound of the heel | NA | Kemp JP, 2017 | ebi-a-GCST006288 |
| Whole body fat free mass | 454,850 | Body composition estimation by impedance measurement. Fat free mass in Kg | MRC-IEU | NA | ukb-b-13354 |
| Appendicular lean mass | 450,243 | Appendicular fat-free mass measured by bioelectrical impedance analysis (BIA) | NA | Pei et al, 2020 | ebi-a-GCST90000025 |
| Handgrip strength(right) | 461,089 | Right hand isometric grip strength, measured using a Jamar J00105 hydraulic hand dynamometer | MRC-IEU | NA | ukb-b-10215 |
| Handgrip strength(left) | 461,026 | Left hand isometric grip strength, measured using a Jamar J00105 hydraulic hand dynamometer | MRC-IEU | NA | ukb-b-7478 |
| Vitamin D | 417,580 | Measured by chemiluminescence immunoassay (CLIA) analysis on a DiaSorin Ltd. LIASON XL | NA | Revez et al, 2020 | ebi-a-GCST90000615 |

**Supplementary Table 2. 109 instrument variables of MS selected for MR analysis**

| SNP | Effect Allele | Other Allele | Effect Allele Frequency | P-value | OR | Beta | SE |
| --- | --- | --- | --- | --- | --- | --- | --- |
| rs10063294 | G | A | 0.429 | 1.13E-09 | 1.104 | 0.099 | 0.016 |
| rs1014486 | C | T | 0.471 | 1.36E-10 | 1.111 | 0.105 | 0.016 |
| rs10191360 | T | C | 0.476 | 3.19E-05 | 1.102 | 0.097 | 0.023 |
| rs10245867 | T | G | 0.323 | 0.00033 | 1.065 | 0.063 | 0.018 |
| rs1026916 | A | G | 0.377 | 1.02E-13 | 1.138 | 0.129 | 0.017 |
| rs10271373 | A | C | 0.497 | 1.65E-05 | 1.073 | 0.070 | 0.016 |
| rs1076928 | T | C | 0.459 | 4.22E-06 | 1.079 | 0.076 | 0.017 |
| rs1077667 | C | T | 0.763 | 8.37E-13 | 1.164 | 0.152 | 0.021 |
| rs10801908 | C | T | 0.868 | 3.54E-16 | 1.240 | 0.215 | 0.026 |
| rs10951042 | C | T | 0.387 | 2.02E-06 | 1.082 | 0.079 | 0.017 |
| rs10951154 | C | T | 0.153 | 6.73E-05 | 1.106 | 0.101 | 0.025 |
| rs11125803 | C | T | 0.252 | 0.001722 | 1.062 | 0.060 | 0.019 |
| rs1112718 | A | G | 0.591 | 2.46E-10 | 1.111 | 0.105 | 0.017 |
| rs11231749 | C | T | 0.296 | 2.34E-05 | 1.076 | 0.073 | 0.017 |
| rs11256593 | T | C | 0.561 | 6.78E-27 | 1.205 | 0.186 | 0.017 |
| rs11578655 | G | T | 0.136 | 0.000482 | 1.094 | 0.090 | 0.026 |
| rs11749040 | A | G | 0.131 | 3.54E-17 | 1.217 | 0.196 | 0.023 |
| rs1177228 | G | A | 0.739 | 8.57E-09 | 1.113 | 0.107 | 0.019 |
| rs11809700 | T | C | 0.277 | 3.51E-15 | 1.155 | 0.144 | 0.018 |
| rs11852059 | C | A | 0.181 | 3.02E-06 | 1.101 | 0.096 | 0.021 |
| rs11899404 | C | T | 0.562 | 0.000757 | 1.109 | 0.103 | 0.031 |
| rs11919880 | A | G | 0.584 | 1.97E-05 | 1.073 | 0.070 | 0.017 |
| rs12147246 | A | G | 0.351 | 4.29E-09 | 1.104 | 0.099 | 0.017 |
| rs12211604 | G | A | 0.384 | 1.86E-07 | 1.098 | 0.093 | 0.018 |
| rs12365699 | G | A | 0.847 | 3.15E-10 | 1.155 | 0.144 | 0.023 |
| rs12434551 | A | T | 0.542 | 1.83E-10 | 1.110 | 0.104 | 0.016 |
| rs12622670 | T | C | 0.550 | 1.04E-10 | 1.113 | 0.107 | 0.017 |
| rs12925972 | C | T | 0.526 | 3.07E-08 | 1.099 | 0.094 | 0.017 |
| rs12971909 | A | G | 0.372 | 8.98E-06 | 1.090 | 0.086 | 0.019 |
| rs13066789 | C | T | 0.447 | 3.17E-05 | 1.072 | 0.070 | 0.017 |
| rs13327021 | T | C | 0.379 | 4.50E-11 | 1.120 | 0.113 | 0.017 |
| rs1365120 | C | T | 0.108 | 5.76E-06 | 1.127 | 0.120 | 0.026 |
| rs1399180 | C | T | 0.818 | 0.000375 | 1.083 | 0.080 | 0.022 |
| rs140522 | T | C | 0.340 | 2.85E-10 | 1.117 | 0.111 | 0.018 |
| rs1465697 | T | C | 0.238 | 3.48E-11 | 1.132 | 0.124 | 0.019 |
| rs17051321 | T | C | 0.216 | 4.95E-07 | 1.099 | 0.094 | 0.019 |
| rs1738074 | C | T | 0.548 | 9.91E-12 | 1.120 | 0.113 | 0.017 |
| rs17724508 | T | C | 0.956 | 2.59E-08 | 1.238 | 0.213 | 0.038 |
| rs17741873 | G | T | 0.809 | 2.15E-05 | 1.093 | 0.089 | 0.021 |
| rs1800693 | C | T | 0.430 | 1.02E-13 | 1.135 | 0.127 | 0.017 |
| rs2084007 | C | T | 0.500 | 1.15E-06 | 1.083 | 0.080 | 0.016 |
| rs2150879 | G | A | 0.441 | 3.29E-10 | 1.109 | 0.103 | 0.016 |
| rs2269434 | C | T | 0.350 | 5.24E-07 | 1.090 | 0.086 | 0.017 |
| rs2286974 | A | G | 0.592 | 1.51E-07 | 1.115 | 0.109 | 0.021 |
| rs2289746 | C | T | 0.646 | 1.48E-06 | 1.089 | 0.085 | 0.018 |
| rs2317231 | G | T | 0.541 | 1.90E-09 | 1.106 | 0.101 | 0.017 |
| rs2469434 | C | T | 0.398 | 0.000309 | 1.063 | 0.061 | 0.017 |
| rs2546890 | A | G | 0.503 | 1.04E-12 | 1.124 | 0.117 | 0.016 |
| rs2585447 | C | T | 0.211 | 7.89E-08 | 1.137 | 0.128 | 0.024 |
| rs2590438 | G | T | 0.331 | 0.001561 | 1.055 | 0.054 | 0.017 |
| rs2836438 | A | G | 0.119 | 2.98E-05 | 1.104 | 0.099 | 0.024 |
| rs28703878 | G | A | 0.309 | 4.51E-10 | 1.143 | 0.134 | 0.021 |
| rs2986736 | C | T | 0.201 | 3.41E-05 | 1.112 | 0.106 | 0.026 |
| rs3184504 | T | C | 0.466 | 3.45E-05 | 1.071 | 0.069 | 0.017 |
| rs32658 | T | G | 0.395 | 2.95E-05 | 1.071 | 0.069 | 0.016 |
| rs34681760 | C | T | 0.643 | 5.28E-06 | 1.084 | 0.081 | 0.018 |
| rs34695601 | T | C | 0.760 | 3.17E-08 | 1.116 | 0.110 | 0.020 |
| rs354033 | G | A | 0.740 | 1.21E-08 | 1.114 | 0.108 | 0.019 |
| rs35486093 | G | A | 0.084 | 1.60E-10 | 1.197 | 0.180 | 0.028 |
| rs35540610 | C | T | 0.224 | 2.88E-12 | 1.145 | 0.135 | 0.019 |
| rs35703946 | G | A | 0.858 | 1.94E-09 | 1.188 | 0.172 | 0.029 |
| rs3737798 | A | G | 0.530 | 1.40E-07 | 1.091 | 0.087 | 0.017 |
| rs3809627 | C | A | 0.571 | 3.25E-08 | 1.102 | 0.097 | 0.018 |
| rs3923387 | T | C | 0.425 | 2.35E-05 | 1.073 | 0.070 | 0.017 |
| rs405343 | T | G | 0.174 | 4.72E-08 | 1.126 | 0.119 | 0.022 |
| rs4325907 | C | T | 0.339 | 3.68E-09 | 1.104 | 0.099 | 0.017 |
| rs438613 | C | T | 0.488 | 9.43E-17 | 1.148 | 0.138 | 0.017 |
| rs4728142 | A | G | 0.451 | 0.000259 | 1.063 | 0.061 | 0.017 |
| rs4796224 | G | A | 0.457 | 1.62E-07 | 1.089 | 0.085 | 0.016 |
| rs55858457 | T | G | 0.376 | 1.20E-08 | 1.120 | 0.113 | 0.020 |
| rs57116599 | G | A | 0.744 | 2.59E-09 | 1.128 | 0.120 | 0.020 |
| rs59655222 | T | C | 0.741 | 3.76E-11 | 1.131 | 0.123 | 0.019 |
| rs6032662 | C | T | 0.252 | 2.85E-13 | 1.143 | 0.134 | 0.018 |
| rs60600003 | G | T | 0.102 | 4.20E-07 | 1.143 | 0.134 | 0.026 |
| rs6072343 | A | G | 0.153 | 0.000891 | 1.079 | 0.076 | 0.023 |
| rs61708525 | G | A | 0.315 | 1.91E-05 | 1.083 | 0.080 | 0.019 |
| rs61863928 | G | T | 0.652 | 5.45E-08 | 1.115 | 0.109 | 0.020 |
| rs62013236 | C | T | 0.846 | 2.07E-06 | 1.133 | 0.125 | 0.026 |
| rs62420820 | A | G | 0.226 | 2.50E-13 | 1.147 | 0.137 | 0.019 |
| rs6496663 | C | A | 0.318 | 2.78E-08 | 1.106 | 0.101 | 0.018 |
| rs6564681 | C | T | 0.311 | 2.23E-07 | 1.098 | 0.093 | 0.018 |
| rs6589706 | A | G | 0.450 | 2.20E-09 | 1.109 | 0.103 | 0.017 |
| rs6670198 | T | C | 0.666 | 2.03E-16 | 1.156 | 0.145 | 0.018 |
| rs67111717 | G | A | 0.342 | 4.96E-07 | 1.100 | 0.095 | 0.019 |
| rs6738544 | C | A | 0.661 | 9.49E-05 | 1.068 | 0.066 | 0.017 |
| rs6837324 | G | A | 0.380 | 3.16E-07 | 1.090 | 0.086 | 0.017 |
| rs6911131 | G | A | 0.061 | 3.09E-05 | 1.157 | 0.146 | 0.035 |
| rs6990534 | G | A | 0.670 | 3.60E-09 | 1.113 | 0.107 | 0.018 |
| rs701006 | G | A | 0.592 | 1.35E-11 | 1.121 | 0.114 | 0.017 |
| rs719316 | T | C | 0.536 | 7.05E-05 | 1.067 | 0.065 | 0.016 |
| rs7260482 | C | A | 0.263 | 1.84E-06 | 1.093 | 0.089 | 0.019 |
| rs72922276 | G | A | 0.902 | 6.88E-06 | 1.141 | 0.132 | 0.029 |
| rs72928038 | A | G | 0.179 | 9.01E-11 | 1.174 | 0.160 | 0.025 |
| rs72989863 | G | A | 0.629 | 7.01E-05 | 1.070 | 0.068 | 0.017 |
| rs73414214 | C | A | 0.904 | 0.000374 | 1.109 | 0.103 | 0.029 |
| rs760517 | C | T | 0.584 | 6.62E-06 | 1.088 | 0.084 | 0.019 |
| rs7731626 | G | A | 0.625 | 2.85E-06 | 1.097 | 0.093 | 0.020 |
| rs7855251 | T | C | 0.731 | 4.23E-08 | 1.116 | 0.110 | 0.020 |
| rs7975763 | T | C | 0.215 | 7.80E-09 | 1.129 | 0.121 | 0.021 |
| rs8062446 | T | C | 0.400 | 8.41E-07 | 1.089 | 0.085 | 0.017 |
| rs9308424 | G | A | 0.698 | 1.34E-06 | 1.096 | 0.092 | 0.019 |
| rs9591325 | T | C | 0.950 | 4.16E-10 | 1.237 | 0.213 | 0.034 |
| rs9610458 | T | C | 0.536 | 4.57E-12 | 1.121 | 0.114 | 0.017 |
| rs962052 | C | T | 0.300 | 0.00021 | 1.070 | 0.068 | 0.018 |
| rs9808753 | G | A | 0.156 | 0.000481 | 1.085 | 0.082 | 0.023 |
| rs9843355 | G | A | 0.838 | 4.73E-10 | 1.143 | 0.134 | 0.021 |
| rs9878602 | T | G | 0.515 | 2.60E-07 | 1.087 | 0.083 | 0.016 |
| rs9955954 | A | G | 0.785 | 1.54E-08 | 1.116 | 0.110 | 0.019 |
| rs9992763 | G | T | 0.451 | 4.51E-08 | 1.094 | 0.090 | 0.016 |

**Supplementary Table 3. Univariable MR and sensitivity analysis: MR results between MS and musculoskeletal outcomes and risk factors**

|  | **MS (exposure) to fracture and confounders (outcome)** | | | | | **Heterogeneity test** | | | | **Horizontal pleiotropy test** | |
| --- | --- | --- | --- | --- | --- | --- | --- | --- | --- | --- | --- |
|  | SNP Number |  | beta | SE | P-value |  | Q | Q df | Q-value |  |  |
| **Musculoskeletal outcomes** |  |  |  |  |  |  |  |  |  |  |  |
| Fracture | 109 | IVW | 0.002 | 0.0007 | **0.001** |  |  |  |  | Egger regression intercept: | -8.50E-07 |
|  |  | MR-egger | 0.002 | 0.002 | 0.383 | MR Egger | 118 | 107 | 0.22 | Standard error: | 0.0003 |
|  |  | Weighted median | 0.002 | 0.001 | 0.051 | IVW | 118 | 108 | 0.241 | Directionality p-value: | 0.997 |
|  |  | Weighted mode | 0.001 | 0.002 | 0.620 |  |  |  |  |  |  |
| Frailty | 152 | IVW | 0.012 | 0.004 | **0.007** |  |  |  |  | Egger regression intercept: | 0.002 |
|  |  | MR-egger | -0.017 | 0.016 | 0.296 | MR Egger | 297.2 | 150 | 9.20E-12 | Standard error: | 0.002 |
|  |  | Weighted median | 0.011 | 0.005 | 0.051 | IVW | 299.1 | 151 | 8.10E-12 | Directionality p-value: | 0.331 |
|  |  | Weighted mode | 0.029 | 0.016 | 0.065 |  |  |  |  |  |  |
| Fall | 109 | IVW | 0.004 | 0.001 | **0.005** | MR Egger | 129.7 | 107 | 0.06731 | Egger regression intercept: | 0.0002 |
|  |  | MR-egger | 0.002 | 0.005 | 0.751 | IVW | 129.7 | 108 | 0.07421 | Standard error: | 0.001 |
|  |  | Weighted median | 0.004 | 0.002 | 0.038 |  |  |  |  | Directionality p-value: | 0.649 |
|  |  | Weighted mode | 0.002 | 0.004 | 0.682 |  |  |  |  |  |  |
| **Predisposing factors** |  |  |  |  |  |  |  |  |  |  |  |
| Heel BMD | 110 | IVW | -0.002 | 0.005 | 0.722 | MR Egger | 274.6 | 108 | 1.74E-16 | Egger regression intercept: | -0.003 |
|  |  | MR-egger | 0.027 | 0.020 | 0.186 | IVW | 280.2 | 109 | 4.97E-17 | Standard error: | 0.002 |
|  |  | Weighted median | 0.010 | 0.005 | 0.057 |  |  |  |  | Directionality p-value: | 0.141 |
|  |  | Weighted mode | 0.017 | 0.010 | 0.083 |  |  |  |  |  |  |
| Whole body fat free mass | 109 | IVW | 0.004 | 0.004 | 0.258 | MR Egger | 857.9 | 107 | 1.61E-117 | Egger regression intercept: | -0.002 |
|  |  | MR-egger | 0.019 | 0.013 | 0.155 | IVW | 868.8 | 108 | 3.81E-119 | Standard error: | 0.001 |
|  |  | Weighted median | 0.003 | 0.002 | 0.230 |  |  |  |  | Directionality p-value: | 0.246 |
|  |  | Weighted mode | 0.003 | 0.005 | 0.577 |  |  |  |  |  |  |
| Appendicular fat free mass | 110 | IVW | 0.006 | 0.007 | 0.402 | MR Egger | 1299 | 108 | 2.34E-203 | Egger regression intercept: | -0.003 |
|  |  | MR-egger | 0.036 | 0.024 | 0.140 | IVW | 1320 | 109 | 6.51E-207 | Standard error: | 0.003 |
|  |  | Weighted median | 0.003 | 0.004 | 0.462 |  |  |  |  | Directionality p-value: | 0.194 |
|  |  | Weighted mode | 0.007 | 0.005 | 0.225 |  |  |  |  |  |  |
| Grip strength (right) | 109 | IVW | -0.004 | 0.003 | 0.183 |  |  |  |  | Egger regression intercept: | -0.002 |
|  |  | MR-egger | 0.011 | 0.009 | 0.237 | MR Egger | 323.1 | 107 | 1.49E-23 | Standard error: | 0.001 |
|  |  | Weighted median | -0.002 | 0.002 | 0.477 | IVW | 330.4 | 108 | 2.23E-24 | Directionality p-value: | 0.124 |
|  |  | Weighted mode | -0.003 | 0.006 | 0.626 |  |  |  |  |  |  |
| Grip strength (left) | 109 | IVW | -0.002 | 0.003 | 0.389 | MR Egger | 331 | 107 | 1.03E-24 | Egger regression intercept: | -0.002 |
|  |  | MR-egger | 0.015 | 0.010 | 0.130 | IVW | 341.4 | 108 | 5.08E-26 | Standard error: | 0.001 |
|  |  | Weighted median | 0.000 | 0.002 | 0.990 |  |  |  |  | Directionality p-value: | 0.069 |
|  |  | Weighted mode | 0.006 | 0.007 | 0.404 |  |  |  |  |  |  |
| Vitamin D | 105 | IVW | -0.003 | 0.003 | 0.427 | MR Egger | 252.9 | 103 | 1.30E-14 | Egger regression intercept: | -0.0002 |
|  |  | MR-egger | -0.001 | 0.012 | 0.925 | IVW | 252.9 | 104 | 2.02E-14 | Standard error: | 0.001 |
|  |  | Weighted median | 0.001 | 0.003 | 0.693 |  |  |  |  | Directionality p-value: | 0.9 |
|  |  | Weighted mode | 0.003 | 0.007 | 0.673 |  |  |  |  |  |  |

IVW, Inverse variance weighted

**Supplementary Table 4. Single SNP MR effects between MS and falls**

| SNP | Beta | SE | P-value |
| --- | --- | --- | --- |
| **rs9878602** | **0.041** | **0.014** | **0.004** |
| **rs7731626** | **0.034** | **0.013** | **0.010** |
| **rs9808753** | **0.051** | **0.021** | **0.015** |
| **rs10191360** | **-0.027** | **0.012** | **0.027** |
| **rs11852059** | **0.035** | **0.016** | **0.028** |
| **rs1026916** | **0.020** | **0.010** | **0.034** |
| **rs701006** | **0.022** | **0.011** | **0.040** |
| **rs140522** | **0.023** | **0.011** | **0.040** |
| **rs3737798** | **0.028** | **0.014** | **0.043** |
| rs11578655 | 0.039 | 0.020 | 0.052 |
| rs9843355 | 0.022 | 0.011 | 0.053 |
| rs12925972 | 0.024 | 0.013 | 0.054 |
| rs6496663 | -0.023 | 0.013 | 0.071 |
| rs3923387 | 0.030 | 0.017 | 0.076 |
| rs2590438 | -0.040 | 0.023 | 0.079 |
| rs35486093 | 0.020 | 0.012 | 0.081 |
| rs11749040 | 0.015 | 0.009 | 0.091 |
| rs6911131 | -0.031 | 0.018 | 0.092 |
| rs9591325 | 0.018 | 0.011 | 0.097 |
| rs11809700 | 0.015 | 0.009 | 0.101 |
| rs17741873 | 0.026 | 0.017 | 0.123 |
| rs6032662 | 0.015 | 0.010 | 0.138 |
| rs9992763 | 0.019 | 0.013 | 0.148 |
| rs11231749 | 0.025 | 0.017 | 0.154 |
| rs1014486 | -0.016 | 0.011 | 0.157 |
| rs719316 | 0.026 | 0.018 | 0.163 |
| rs62420820 | -0.014 | 0.010 | 0.168 |
| rs10063294 | 0.016 | 0.012 | 0.170 |
| rs61863928 | -0.016 | 0.011 | 0.175 |
| rs13327021 | 0.015 | 0.011 | 0.182 |
| rs2269434 | 0.018 | 0.014 | 0.206 |
| rs8062446 | -0.018 | 0.014 | 0.212 |
| rs9610458 | -0.013 | 0.010 | 0.217 |
| rs55858457 | 0.013 | 0.011 | 0.231 |
| rs2836438 | -0.021 | 0.017 | 0.236 |
| rs10245867 | -0.022 | 0.020 | 0.266 |
| rs405343 | 0.014 | 0.013 | 0.276 |
| rs35540610 | 0.011 | 0.010 | 0.290 |
| rs61708525 | 0.017 | 0.016 | 0.290 |
| rs4796224 | -0.014 | 0.014 | 0.297 |
| rs73414214 | -0.021 | 0.020 | 0.310 |
| rs10271373 | 0.017 | 0.017 | 0.312 |
| rs1077667 | -0.009 | 0.010 | 0.323 |
| rs12365699 | -0.011 | 0.011 | 0.331 |
| rs32658 | 0.016 | 0.017 | 0.345 |
| rs6072343 | 0.021 | 0.022 | 0.356 |
| rs12147246 | -0.011 | 0.012 | 0.368 |
| rs2286974 | 0.010 | 0.011 | 0.370 |
| rs438613 | 0.008 | 0.009 | 0.372 |
| rs72922276 | -0.012 | 0.014 | 0.389 |
| rs9308424 | -0.012 | 0.014 | 0.390 |
| rs6589706 | -0.010 | 0.011 | 0.393 |
| rs2469434 | 0.017 | 0.020 | 0.395 |
| rs1800693 | -0.008 | 0.009 | 0.396 |
| rs1399180 | 0.016 | 0.020 | 0.420 |
| rs10801908 | 0.007 | 0.008 | 0.432 |
| rs1177228 | -0.010 | 0.012 | 0.441 |
| rs10951154 | 0.013 | 0.018 | 0.452 |
| rs1365120 | -0.011 | 0.015 | 0.457 |
| rs2084007 | 0.011 | 0.015 | 0.470 |
| rs1465697 | -0.008 | 0.011 | 0.492 |
| rs6564681 | -0.009 | 0.014 | 0.492 |
| rs4325907 | -0.008 | 0.012 | 0.501 |
| rs72989863 | -0.012 | 0.018 | 0.504 |
| rs4728142 | 0.013 | 0.019 | 0.504 |
| rs2150879 | -0.008 | 0.011 | 0.511 |
| rs6670198 | 0.005 | 0.009 | 0.525 |
| rs354033 | 0.008 | 0.013 | 0.529 |
| rs2986736 | 0.008 | 0.014 | 0.555 |
| rs12971909 | 0.008 | 0.014 | 0.572 |
| rs60600003 | 0.008 | 0.015 | 0.586 |
| rs2546890 | 0.005 | 0.010 | 0.599 |
| rs1112718 | 0.006 | 0.011 | 0.618 |
| rs11899404 | 0.006 | 0.012 | 0.636 |
| rs962052 | 0.009 | 0.020 | 0.639 |
| rs34695601 | 0.006 | 0.012 | 0.647 |
| rs7975763 | 0.005 | 0.012 | 0.650 |
| rs57116599 | -0.005 | 0.012 | 0.657 |
| rs7855251 | -0.006 | 0.012 | 0.657 |
| rs6990534 | 0.005 | 0.012 | 0.667 |
| rs67111717 | 0.006 | 0.013 | 0.670 |
| rs12622670 | -0.005 | 0.011 | 0.671 |
| rs28703878 | 0.004 | 0.010 | 0.697 |
| rs2585447 | -0.005 | 0.013 | 0.717 |
| rs1738074 | -0.004 | 0.010 | 0.718 |
| rs13066789 | -0.006 | 0.017 | 0.723 |
| rs2289746 | 0.005 | 0.015 | 0.731 |
| rs2317231 | 0.004 | 0.012 | 0.732 |
| rs11919880 | 0.006 | 0.017 | 0.741 |
| rs11256593 | -0.002 | 0.006 | 0.755 |
| rs1076928 | 0.004 | 0.016 | 0.786 |
| rs1250551 | -0.003 | 0.011 | 0.794 |
| rs3184504 | -0.004 | 0.017 | 0.796 |
| rs6837324 | 0.004 | 0.014 | 0.798 |
| rs6738544 | -0.005 | 0.018 | 0.804 |
| rs72928038 | -0.001 | 0.010 | 0.891 |
| rs17051321 | -0.001 | 0.015 | 0.921 |
| rs34681760 | -0.002 | 0.016 | 0.924 |
| rs10951042 | -0.001 | 0.015 | 0.931 |
| rs7260482 | -0.001 | 0.015 | 0.942 |
| rs62013236 | 0.001 | 0.014 | 0.969 |
| rs59655222 | 0.000 | 0.011 | 0.973 |
| rs17724508 | 0.000 | 0.012 | 0.973 |
| rs11125803 | 0.001 | 0.022 | 0.973 |
| rs9955954 | -0.001 | 0.041 | 0.977 |
| rs35703946 | 0.000 | 0.010 | 0.981 |
| rs12211604 | 0.000 | 0.013 | 0.990 |
| rs760517 | 0.000 | 0.016 | 0.994 |
| rs3809627 | 0.000 | 0.012 | 0.998 |
| All - Inverse variance weighted | 0.004 | 0.001 | 0.005 |
| All - MR Egger | 0.002 | 0.005 | 0.751 |

| **Supplementary Table 5. Information of significant single SNPs in MR between MS and falls** | | | | | |  | |
| --- | --- | --- | --- | --- | --- | --- | --- |
|  | | | |  | |  | |
| SNP | SNP function | Gene | Diseases related with gene | | Pathway | |  |
| rs9878602 | Intron Variant | FOXP1 (Forkhead Box P1) | Intellectual Developmental Disorder With Language Impairment | | Nervous system development  Wnt / Hedgehog / Notch | |  |
| rs7731626 | genic_upstream_transcript  _variant,intron_variant | ANKRD55 (Ankyrin Repeat Domain 55) | Oligoarticular Juvenile Idiopathic Arthritis and Rheumatoid Factor-Negative Polyarticular Juvenile Idiopathic Arthritis | |  | |  |
| rs9808753 | missense_variant,coding  _sequence_variant | IFNGR2 (Interferon Gamma Receptor 2) | Immunodeficiency 28 and Autosomal Dominant Mendelian Susceptibility To Mycobacterial Diseases | | IL27-mediated signaling events and Interferon gamma signaling | |  |
| rs10191360 | intron_variant,genic  _upstream_transcript_variant | GNG2 (G Protein Subunit Gamma 2) | Familial Hemiplegic Migraine | | Signaling by Slit and Thyroid stimulating hormone (TSH) signaling pathway | |  |
| rs11852059 | intron_variant,geni  c_upstream_transcript_variant | GNG2 (G Protein Subunit Gamma 2) | Familial Hemiplegic Migraine | | Signaling by Slit and Thyroid stimulating hormone (TSH) signaling pathway | |  |
| rs1026916 | intron_variant | STAT3 (Signal Transducer  And Activator Of Transcription 3) | Autoimmune Disease, Multisystem, Infantile-Onset, 1 and Hyper-Ige Recurrent Infection Syndrome 1 | | Cellular Senescence and 4-1BB Pathway | |  |
| rs701006 | intron_variant | OS9 (OS9 Endoplasmic Reticulum Lectin) | Bone Cancer and  Rhabdomyosarcoma | | Translational Control and Regulation of activated PAK-2p34 by proteasome mediated degradation | |  |
| rs140522 | upstream_transcript_variant,  2KB_upstream_variant | ODF3B (Outer Dense Fiber  Of Sperm Tails 3B) | NA | | NA | |  |
| rs3737798 | intron_variant,3_prime_UTR_variant | VANGL2 (VANGL Planar Cell Polarity Protein 2) | Neural Tube Defects and Isolated Exencephaly | | RND3 GTPase cycle and Regulation of activated PAK-2p34 by proteasome mediated degradation | |  |

**Supplementary Table 6. Multivariable MR between MS and fracture adjusting for predisposing factors**

|  | MR effects of MS to fracture | | | | |
| --- | --- | --- | --- | --- | --- |
|  | Beta | SE | P-value | OR | 95% CI |
| **Unadjusted model (MR between MS and fracture)** | **0.002** | **0.001** | **0.001** | **1.002** | **1.000-1.004** |
| **Adjusted by** |  |  |  |  |  |
| frailty | 0.002 | 0.001 | 0.006 | 1.002 | 1.000-1.004 |
| fall | 0.002 | 0.001 | 0.007 | 1.002 | 1.000-1.004 |
| Appendicular fat free mass | 0.001 | 0.001 | 0.025 | 1.001 | 0.999-1.003 |
| whole body fat free mass | 0.002 | 0.001 | 0.01 | 1.002 | 1.000-1.004 |
| heel BMD | 0.002 | 0.001 | 0.013 | 1.002 | 1.000-1.004 |
| vitamin D | 0.002 | 0.001 | 0.002 | 1.002 | 1.000-1.004 |
| right grip strength | 0.002 | 0.001 | 0.002 | 1.002 | 1.000-1.004 |
| left grip strength | 0.002 | 0.001 | 0.001 | 1.002 | 1.000-1.004 |
| frailty+ fall | 0.002 | 0.001 | 0.012 | 1.002 | 1.000-1.004 |
| frailty+ fall+ heel BMD+whole body fat free mass + appendicular fat free mass** | 0.002 | 0.001 | 0.024 | 1.002 | 1.000-1.004 |
| heel BMD+whole body fat free mass + appendicular fat free mass+ vitd+gripR+gripL | 0.002 | 0.001 | 0.004 | 1.002 | 1.000-1.004 |
| frailty+ fall+ heel BMD+whole body fat free mass + appendicular fat free mass+vitd+gripR+gripL | 0.002 | 0.001 | 0.033 | 1.002 | 1.000-1.004 |

**Supplementary Table 7. Multivariable MR between MS and frailty adjusting for predisposing factors**

|  | MR effects of MS to frailty | | | | |
| --- | --- | --- | --- | --- | --- |
|  | Beta | SE | P-value | OR | 95% CI |
| **Unadjusted model** **(MR between MS and frailty)** | **0.012** | **0.004** | **0.007** | **1.012** | **1.004-1.020** |
| Adjusted by fall | 0.002 | 0.001 | 0.058 | 1.002 | 1.000-1.004 |
| Adjusted by  appendicular fat free mass | 0.013 | 0.004 | 0.003 | 1.013 | 1.005-1.021 |
| Adjusted by  whole body fat free mass | 0.013 | 0.004 | 0.004 | 1.013 | 1.005-1.021 |
| Adjusted by right grip strength | 0.011 | 0.005 | 0.02 | 1.011 | 1.001-1.021 |
| Adjusted by left grip strength | 0.011 | 0.004 | 0.015 | 1.011 | 1.003-1.019 |
| Adjusted by all except fall* | 0.013 | 0.005 | 0.004 | 1.013 | 1.003-1.023 |
| Adjusted by all | 0.01 | 0.004 | 0.027 | 1.01 | 1.002-1.018 |

**Supplementary Table 8. Colocalization analysis for 9 significant SNPs in MR analysis between MS and Falls**

| SNP | Position* | Posterior probability of colocalization (overall PP.H4) | SNP probability of colocalization  (Single SNP PP.H4) |
| --- | --- | --- | --- |
| rs9878602 | 3:71486187 | 0.031 | 0.067 |
| **rs7731626** | **5:55444683** | **0.005** | **0.927** |
| rs9808753 | 21:34787312 | 0.002 | 0.024 |
| rs10191360 | 2:136884679 | 0.001 | 0.162 |
| rs11852059 | 14:52306091 | 0.006 | 0.059 |
| rs1026916 | 17:40529835 | 0.006 | 0.062 |
| rs701006 | 12:58106836 | 0.282 | 0.208 |
| **rs140522** | **22:50971266** | **0.005** | **0.927** |
| rs3737798 | 1:160389984 | 0.003 | 0.482 |

*GRCh37


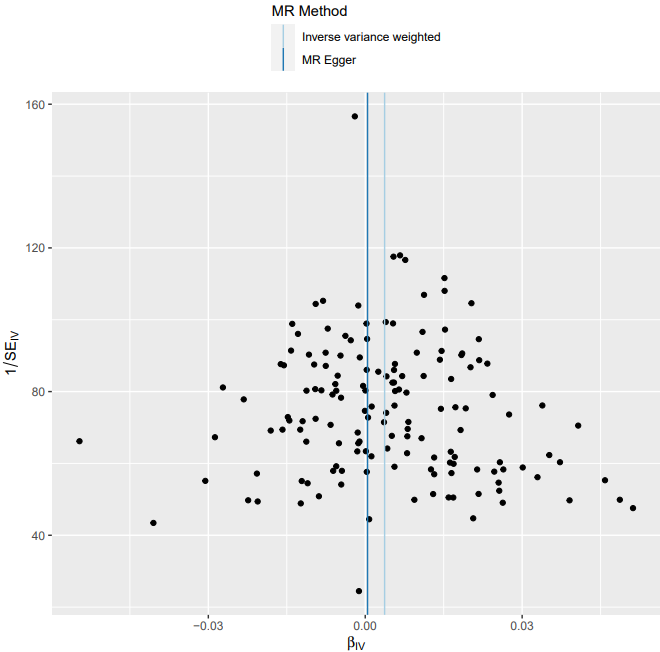


**Supplementary Figure 1. Funnel plot of MR results between MS and falls**
